# Non-invasive ultrasound localization microscopy (ULM) in azoospermia: connecting testicular microcirculation to spermatogenic functions

**DOI:** 10.7150/thno.99668

**Published:** 2024-08-19

**Authors:** Maoyao Li, Xingxuan Zhang, Jipeng Yan, Huiquan Shu, Zitong Li, Chujun Ye, Lei Chen, Chao Feng, Yuanyi Zheng

**Affiliations:** 1 Department of Ultrasound in Medicine, Shanghai Sixth People's Hospital Affiliated to Shanghai Jiao Tong University School of Medicine, Yishan Road 600, Shanghai, 200233, China.; 2 Department of Bioengineering, Imperial College London, Exhibition Road, London, SW7 2AZ, U.K.; 3 Department of Reproductive Medicine, The International Peace Maternity and Child Health Hospital, School of Medicine, Shanghai Jiao Tong University, Shanghai, 200030, China.; 4 Shanghai Key Laboratory of Embryo Original Disease, Shanghai 200030, China.

**Keywords:** ultrasound localization microscopy (ULM), pressure distribution maps, testicular microcirculation, spermatogenic functions, azoospermia

## Abstract

**Rationale:** Azoospermia is a significant reproductive challenge. Differentiating between non-obstructive azoospermia (NOA) and obstructive azoospermia (OA) is crucial as each type requires distinct management strategies. Testicular microcirculation plays a profound role in spermatogenic functions. However, current diagnostic methods are limited in their ability to effectively elucidate this crucial connection.

**Methods:** We employed ultrasound localization microscopy (ULM) to visualize testicular microcirculation in NOA and OA patients and quantified the testicular hemodynamic parameters. Pearson correlation analysis was conducted to investigate the inner connection between parameters of testicular microcirculation and clinical spermatogenic functions. We conducted multiple logistic regression analysis to establish a new diagnostic model that integrates follicle-stimulating hormone (FSH) and mean vascular diameter to distinguish NOA from OA.

**Results:** Our findings demonstrated significant differences in vascular parameters between NOA and OA, with NOA characterized by lower mean vascular diameter (p < 0.001), vessel density (p < 0.001), and fractal number (p < 0.001). Testicular volume showed a moderate positive correlation with mean vascular diameter (r = 0.419, p < 0.01) and vessel density (r = 0.415, p < 0.01); Mean vascular diameter exhibited negative correlations with both FSH (r = -0.214, p < 0.05) and age (r = -0.240, p < 0.05); FSH (r = -0.202, p < 0.05) and luteinizing hormone (LH) (r = -0.235, p < 0.05) were negatively correlated with mean blood flow velocity. The diagnostic model demonstrated an area under the curve (AUC) of 0.968. We also reported a method to map the vascular pressure distribution derived from the blood flow velocity generated by ULM.

**Conclusions:** ULM provides a non-invasive and detailed assessment of testicular microvascular dynamics. The ULM-derived vascular parameters are able to connect testicular microcirculation to spermatogenic functions. The combination of FSH and mean vascular diameter enhances diagnostic precision and holds potential for distinguishing NOA from OA.

## Introduction

Azoospermia affects approximately 1% of all men and 10-15% of those with infertility, representing a severe reproductive hurdle 1, 2. Clinically distinguishing between non-obstructive azoospermia (NOA) and obstructive azoospermia (OA) is paramount, as each type requires distinct management strategies. OA generally allows for straightforward surgical remedies that can restore fertility, whereas NOA, linked to intrinsic testicular failure, might necessitate more complex interventions such as assisted reproductive technologies 3, 4. Current clinical methods for differentiating NOA from OA face significant limitations. As the golden standard, testicular biopsy provides detailed insights but is invasive and carries risks like infection and potential damage to testicular tissue, which could further impair fertility 5, 6. Hormonal tests often lack specificity and can be influenced by other health conditions, reducing their reliability in providing a definitive diagnosis 7, 8. Moreover, conventional imaging techniques like ultrasound, although routinely used, fail to detect microstructural changes within the testes essential for accurate diagnosis 9, 10.

The critical role of testicular microcirculation in maintaining normal testicular functions is well-established. In the human testis, capillaries form a sequential link between Leydig cells and seminiferous tubules, facilitating hormone transport and nutrient delivery essential for spermatogenesis 11, 12. Despite this, there remains a significant deficiency in correlating testicular microcirculation dynamics with spermatogenic functions. This deficiency hinders our ability to fully comprehend how alterations in microcirculation can impact effective spermatogenesis and vice versa.

Therefore, uncovering the inner connection between testicular microcirculation and spermatogenic functions is crucial for improving diagnosis and treatment of male infertility and its associated disorders. Ultrasound localization microscopy (ULM) represents a significant advancement in the visualization of microvascular structures, surpassing the limitations of traditional ultrasound imaging by resolving features below the diffraction limit of acoustic wave 13-15. This technology has been widely applied in fields such as oncology, neurology, and cardiology, where detailed microvascular assessments are essential 16-25. Besides, the inherent nature of ULM enables it not only to provide superior image resolution but also to facilitate quantitative assessments of vascular dynamics, such as blood flow velocity and vessel diameter. These quantitative parameters are invaluable for investigating testicular microcircuzlation, enabling the establishment of its underlying connection with spermatogenic functions.

In this study, we utilized ULM to visualize and analyze the testicular microcirculation in cases of NOA and OA, obtaining super-resolution (SR) images that include blood flow direction maps, velocity maps, and, for the first time, pressure distribution maps. By quantifying hemodynamic parameters from ULM, we uncovered the underlying connection between testicular microcirculation and spermatogenic functions. A new diagnostic model that effectively differentiates between NOA and OA was established by the integration of a clinical indicator of spermatogenic functions and a ULM-derived microcirculatory parameter.

## Methods

### Clinical Data Acquisition

The study included 94 infertile outpatients diagnosed with azoospermia, recruited consecutively from Shanghai Sixth People's Hospital and the International Peace Maternity and Child Health Hospital from October 2021 to April 2024. Patients with any of the following conditions were excluded: active genital tract infections, malignancies, or recent exogenous hormone administration. Eligible patients underwent a comprehensive evaluation, which included a detailed medical history, semen analysis, physical examination, serum hormonal profile assessment, testicular ultrasound and diagnostic testicular biopsy. The serum hormone tests include follicle-stimulating hormone (FSH), luteinizing hormone (LH), and testosterone (T). Testicular volume was calculated by the formula of Lambert: length × height × width × 0.71 26. Genetic testing for karyotype and Y chromosome microdeletion was performed when necessary. Based on the findings, the patients were diagnosed as NOA or OA. The diagnoses were made according to WHO criteria 27 and guidelines of azoospermia 3. A total of 36 NOA and 58 OA patients were included in this study. This retrospective study was conducted in accordance with the declaration of Helsinki and received approval from the institutional review board (ChiCTR2100048361). Informed consent was obtained from all patients.

### Ultrasound Evaluation Protocol

Initially, representative static images of the bilateral testes were acquired using an L14-3WU linear array probe (Eagus R9, Mindray) with settings of 3.0 cm depth, 70 gain, and 130 dynamic range, following a B-mode scan of the targeted region. Subsequently, the color Doppler flow imaging (CDFI) mode was employed to visualize the main testicular blood flow. Afterward, contrast-enhanced ultrasound (CEUS) dataset was obtained for ULM with the same probe (MI: 0.080; depth: 3.0 cm; gain: 56; dynamic range: 105) to assess the overall distribution of testicular vascular structure.

For the CEUS procedure, 2.4 mL of contrast microbubbles (MBs) (SonoVue) were administered intravenously into a radial vein, immediately followed by a 5 mL saline flush (0.9% saline). The contrast agent reached the testes within approximately 20 seconds, with MBs remaining visible for 2-3 minutes. The MB suspension was prepared strictly according to the manufacturer's instructions. Continuous side-by-side B-mode and CEUS images of the testes were acquired at a frame rate of 114 Hz for 30 seconds, with the data recorded for subsequent ULM analysis.

Ultrasound examinations were conducted by an experienced sonographer with over 20 years of expertise in testicular ultrasound imaging. Probes were positioned to ensure that all testes were scanned through the maximum transverse plane. For safety reasons, all subjects were kept under close medical supervision during and for at least 30 minutes after the administration of SonoVue to monitor for potential serious hypersensitivity reactions.

### Ultrasound Localization Microscopy Processing and Image Quantification

**Figure 1:**
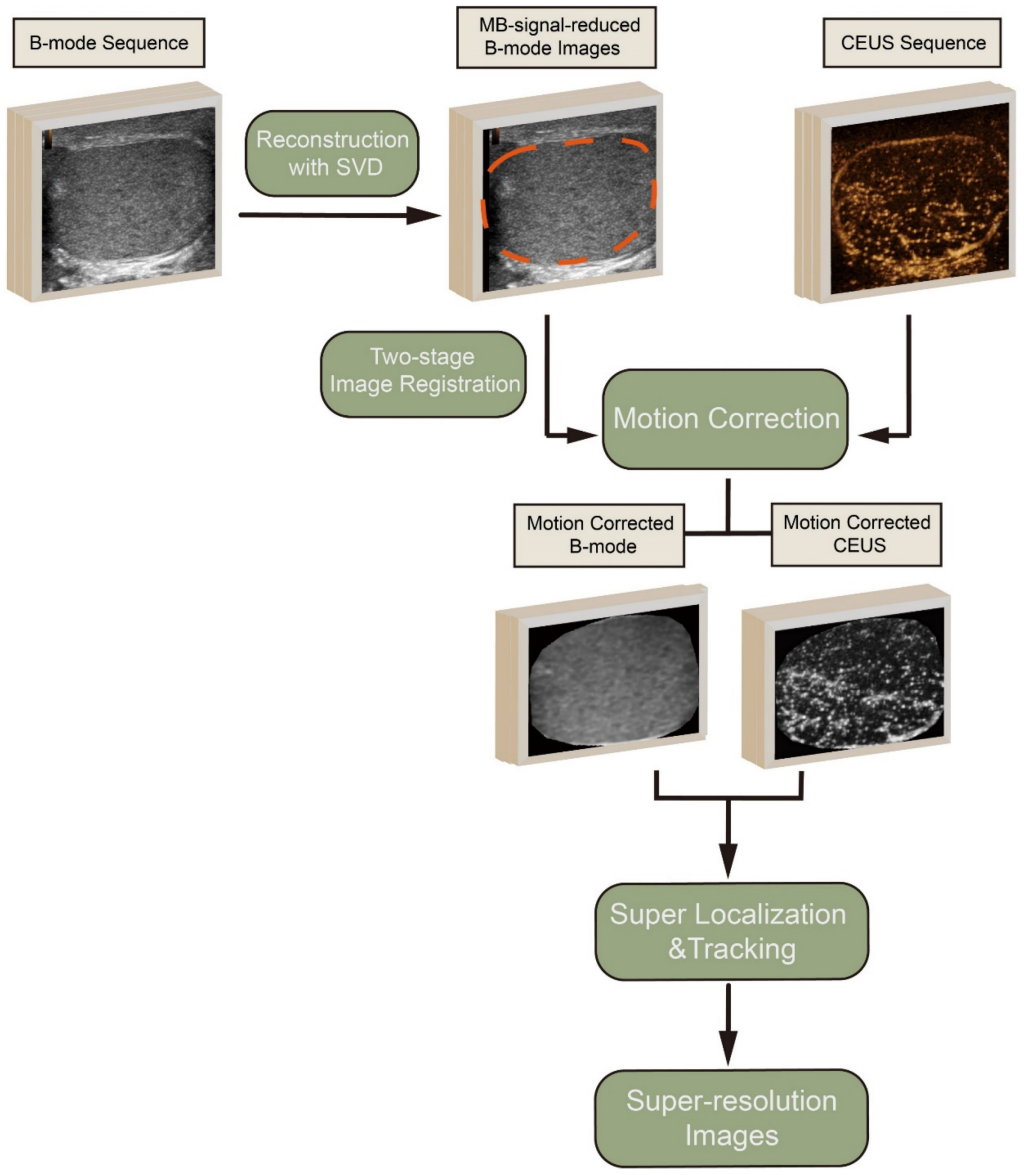
The processing pipeline for generating super-resolution images from contrast-enhanced ultrasound (CEUS) data. The process includes singular value decomposition (SVD), two-stage image registration, motion correction, and super localization and tracking to produce high-quality super-resolution images. MB: microbubble.

The provided diagram illustrates the processing pipeline for generating SR images from CEUS data (Figure 1). The process began with the acquisition of B-mode and CEUS sequences. Tissue motions, which may be induced by breathing or probe pressure, within the region of interest (ROI), were detected on the B-mode images by a two-stage image registration algorithm 28 and corrected for the corresponding CEUS frames. MB signals in B-mode images that affected tissue motion detection were reduced via the reconstruction by discarding small singular values in the singular value decomposition (SVD) of B-mode sequence.

In the subsequent step, super localization and tracking of MBs were performed using a framework proposed in our previous work 20, 29. This involves identifying and tracking individual MBs within the CEUS sequence. MBs that were identified to exist in fewer than three frames by tracking were discarded to further reduce noise.

The movement trajectories of the MBs were formed by linking each pair of MBs with lines. To create the high-quality SR MB density map, these trajectories were accumulated on the respective pixels and then blurred using a two-dimensional Gaussian filter. The standard deviation (SD) of the filter was set according to the localization uncertainty of the MBs. Flow velocity for each pixel was determined by the vector mean of the passed trajectories. Accompanying the MB density map, the MB flow magnitude and direction were represented in two separate images.

The SR images were quantified using several parameters, including flow velocity, tortuosity, diameter, fractal number and vessel density. Flow velocity's mean and SD were derived from the non-zero values present in the flow magnitude map. Tortuosity was measured by dividing the total length of each track by the direct distance between its endpoints. By applying a threshold set at half the peak value of the two-dimensional Gaussian filter used for blurring, a binary vessel structure was obtained from the MB density. The 'bwmorph' function was used to estimate the centerlines of the vessel structures, and vessel diameter was defined as twice the closest distance from any point on these centerlines to the vessel boundary. Fractal number quantifies the complexity of vasculature as a ratio of the change in details to the change in scale, describing the self-similarity of vasculature across different scales. Fractal analysis was performed using the box counting method. The SR images were divided into squares of varying scales, and the number of squares containing vascular features at each scale was counted. The fractal number was subsequently obtained by averaging the local slopes in the semi-log plot, which remained almost constant within a certain range. The area ratio of the vessel structure to the entire ROI was used to calculate vessel density.

On the basis of above ULM pipeline, we innovatively generated a pressure distribution map. Inspired by the methodology used to calculate pulmonary artery pressure 30, we applied the simplified Bernoulli equation to determine the pressure distribution within the microvasculature. In the simplified Bernoulli equation, pressure is directly proportional to the square of the speed. Based on the blood flow velocity generated by ULM, we obtained the pressure in the microcirculation. After normalization, we plotted the isobars, resulting in the final pressure distribution map.

### Statistical Analysis

ULM processing was performed using the software at https://github.com/JipengYan1995/SRUSSoftware. Data were analyzed using GraphPad Prism 10.1.2. The differences in hemodynamic parameters among patient groups were assessed using the Student's t-test. Pearson correlation analysis was employed to assess the linear relationship between the variables under investigation. The assumptions of linearity, normality, and homogeneity of variances were confirmed through scatterplots, the Shapiro-Wilk test, and the Levene's test respectively.

The velocity histogram represented the average velocity distribution of microvasculature across all patients. Firstly, the ratio of blood vessels with different flow velocities to the total number of vessels was calculated for each patient's velocity chart. Then, the average distribution was obtained for all NOA and OA patients. The same calculation method was used for the tortuosity histogram and the vascular width histogram. Specifically, the tortuosity distribution was calculated as the ratio of trajectories with different tortuosity to the total number of trajectories, and the vascular width distribution was calculated as the ratio of vessels with different widths to the total number of vessels.

The diagnostic performances of the diagnostic models were assessed by the area under the receiver operating characteristic (ROC) curve. Using the optimal cutoff value based on the maximum Youden index, sensitivity and specificity were obtained. To explore the relationship between a binary dependent variable and two independent variables, we conducted multiple logistic regression analysis. Multicollinearity among the independent variables was assessed using the variance inflation factor and was found to be within acceptable limits. The relationship between continuous independent variables and the logit of the dependent variable was confirmed to be linear.

A p-value of < 0.05 was considered statistically significant, and p-values of < 0.01 or < 0.001 were considered highly statistically significant for all the aforementioned tests.

## Results

### Routine Testicular Ultrasound Imaging of Azoospermia Patients

**Figure 2:**
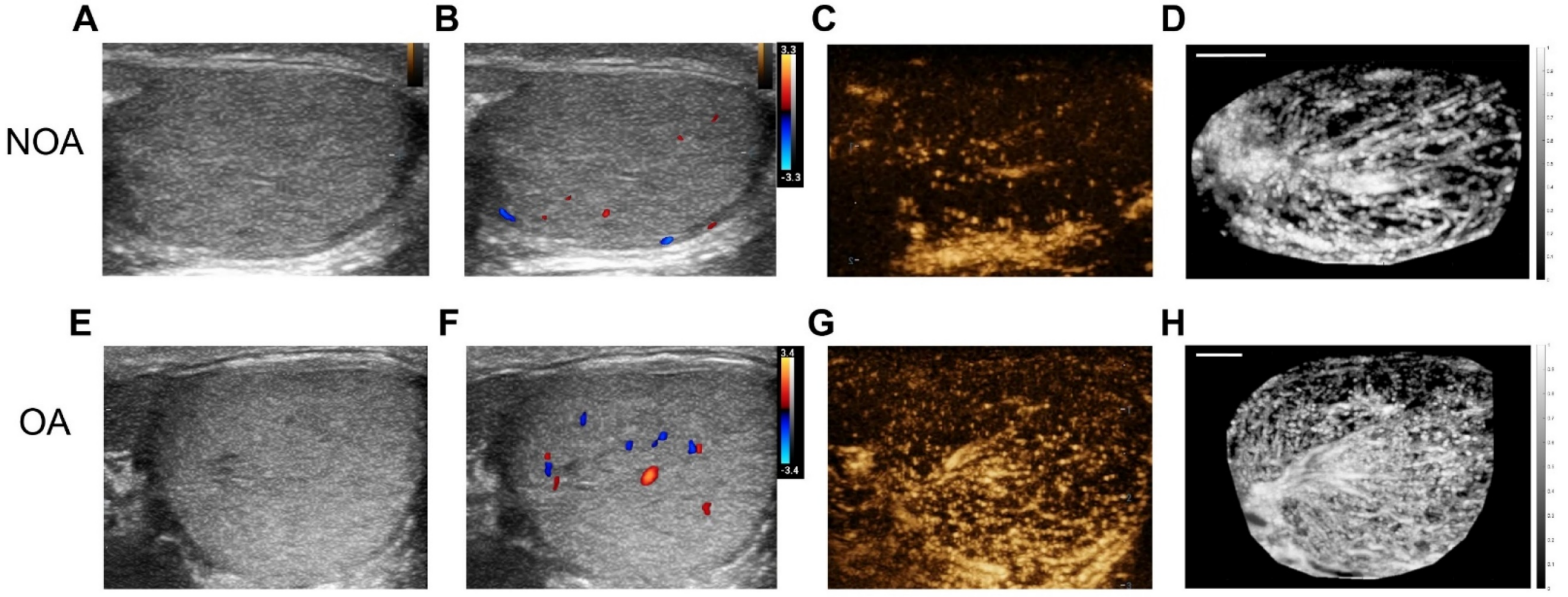
Comparative ultrasonographic assessment of human testes in azoospermia patients. The top row shows images from a patient with non-obstructive azoospermia (NOA), and the bottom row depicts images from a patient with obstructive azoospermia (OA). From left to right: (A) & (E) B-mode ultrasound, (B) & (F) color Doppler flow imaging (CDFI), (C) & (G) contrast-enhanced ultrasound (CEUS), and (D) & (H) maximum intensity projection (MIP) images. To facilitate an intuitive comparison, the NOA images were horizontally mirrored. Scale bars = 5 mm.

We collected a total of 36 NOA and 58 OA patients. Bilateral testicular ultrasound imaging, CDFI, and CEUS were performed on them. Figure 2 presents B-mode imaging, CDFI, a frame from CEUS imaging at the peak of MB perfusion, and maximum intensity projection (MIP) images for a patient with NOA and a patient with OA. CDFI highlighted blood flow within the testes, though it provided limited blood flow information, with sparse dot-like flow signals. The real-time CEUS imaging enhanced the visualization of testicular vascularization. To intuitively display the testicular vascular network, an MIP image was generated by superimposing the highest value pixel of the MB extracted from the CEUS sequence. MIP images offer a comprehensive view of the vascular architecture and morphology of testicular circulation.

From the images, it was observed that for both NOA and OA patients, B-mode images showed the homogeneous texture of the testicular parenchyma. CDFI revealed that the OA patient exhibited slightly richer testicular blood flow signals compared to the NOA patient. The frames from CEUS imaging at the peak of MB perfusion showed that testicular vascular perfusion in NOA was sparser compared to that in OA. MIP images indicate that the testicular vasculature in OA is homogeneously distributed, whereas in NOA, there are regional variations in vessel density, indicating areas of inferior or superior perfusion rather than uniform testicular vascularization.

### Ultrasound Localization Microscopy of Azoospermia Patients

**Figure 3:**
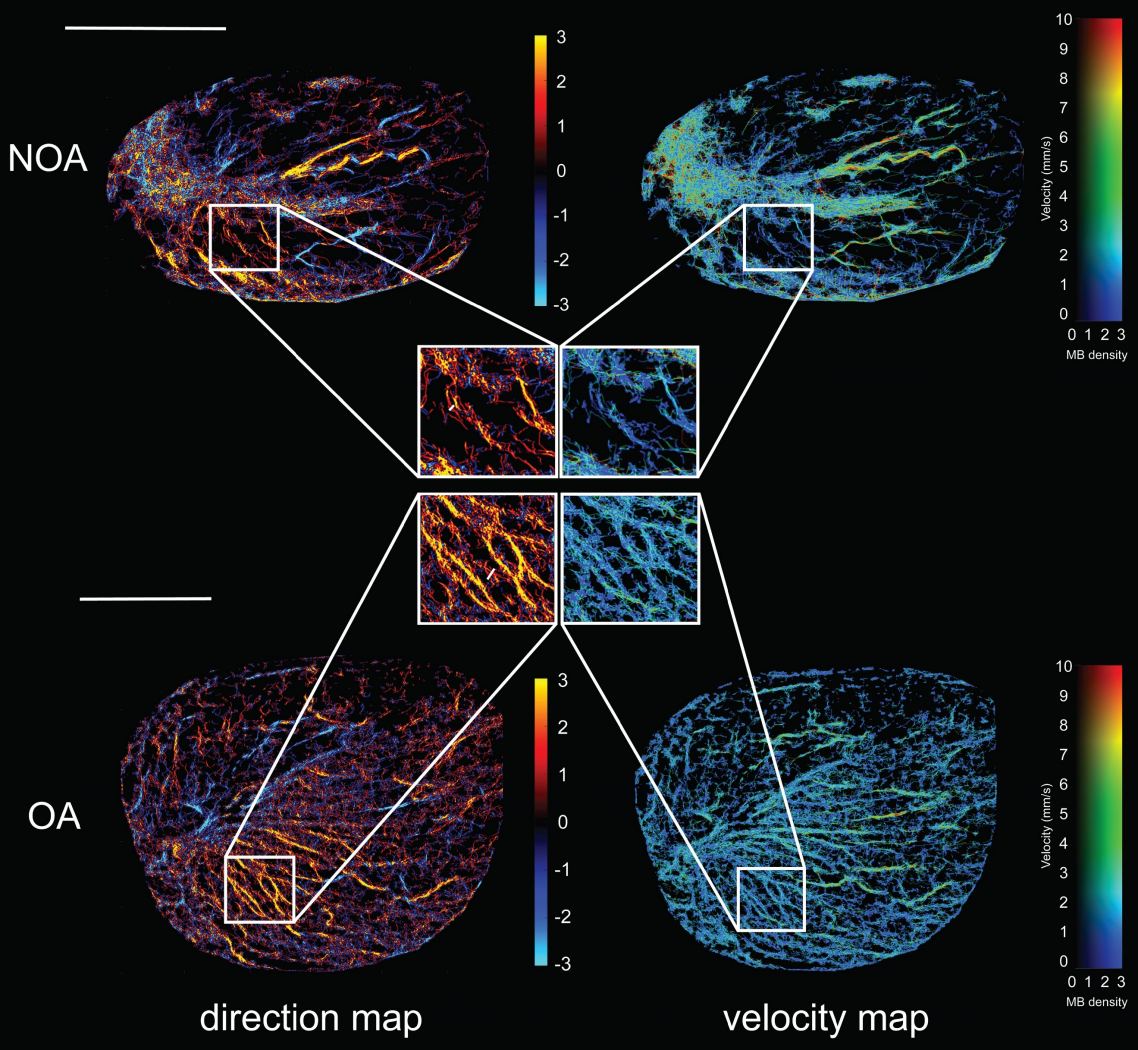
Ultrasound localization microscopy (ULM) of testicular microvasculature in patients with non-obstructive azoospermia (NOA) and obstructive azoospermia (OA). The top panel represents NOA and the bottom panel represents OA. Each panel includes a direction map on the left, displaying the orientation of blood flow (red indicates microbubble flow toward the transducer, while blue signifies flow away from the transducer), and a velocity map on the right, showing the speed of blood flow within the testicular tissue. Highlighted regions of interest (ROIs) within white boxes were magnified to provide a closer view of the vascular patterns and flow dynamics characteristic of each condition. MB: microbubble. Scale bars = 1 cm.

Based on the collected CEUS sequences, we conducted the ULM processing as shown in Figure 1 on all included azoospermia patients. Figure 3 presents high-quality SR ultrasound images of testicular microvasculature for the same NOA and OA patients as shown in Figure 2. By determining the centroid positions of the MBs and tracking them across successive frames, we generated density images by accumulating the MB trajectories on the respective pixels. Flow velocity for each pixel was determined by the mean velocity vector of the passed trajectories. Accompanying the MB density map, the MB flow magnitude and direction were represented in two separate images. The SR images revealed that, compared to OA, NOA was characterized by sparser blood perfusion, lower vascular density, and areas of poor perfusion appearing as darker regions, consistent with observations made in CEUS. In comparison to CDFI, these SR images provided significantly richer microvascular information and finer details. We superimposed the ULM SR images of NOA and OA patients with their MIP images (Figure S1) and found that, compared to MIP images, ULM improved spatial resolution, providing a clearer depiction of the testicular microvasculature. The SR images achieved resolution beyond the wave diffraction limit. However, the spatial resolution of MIP images remained the same as in their CEUS sequence because they were generated by superimposing the maximum values of CEUS at each pixel over time. Additionally, SR images offered the advantage of intuitively displaying hemodynamic information, including blood flow direction and velocity. This was because the MIP images did not involve the localization and tracking of MBs. They depicted the blood vessel morphology but did not provide hemodynamic information.

**Figure 4:**
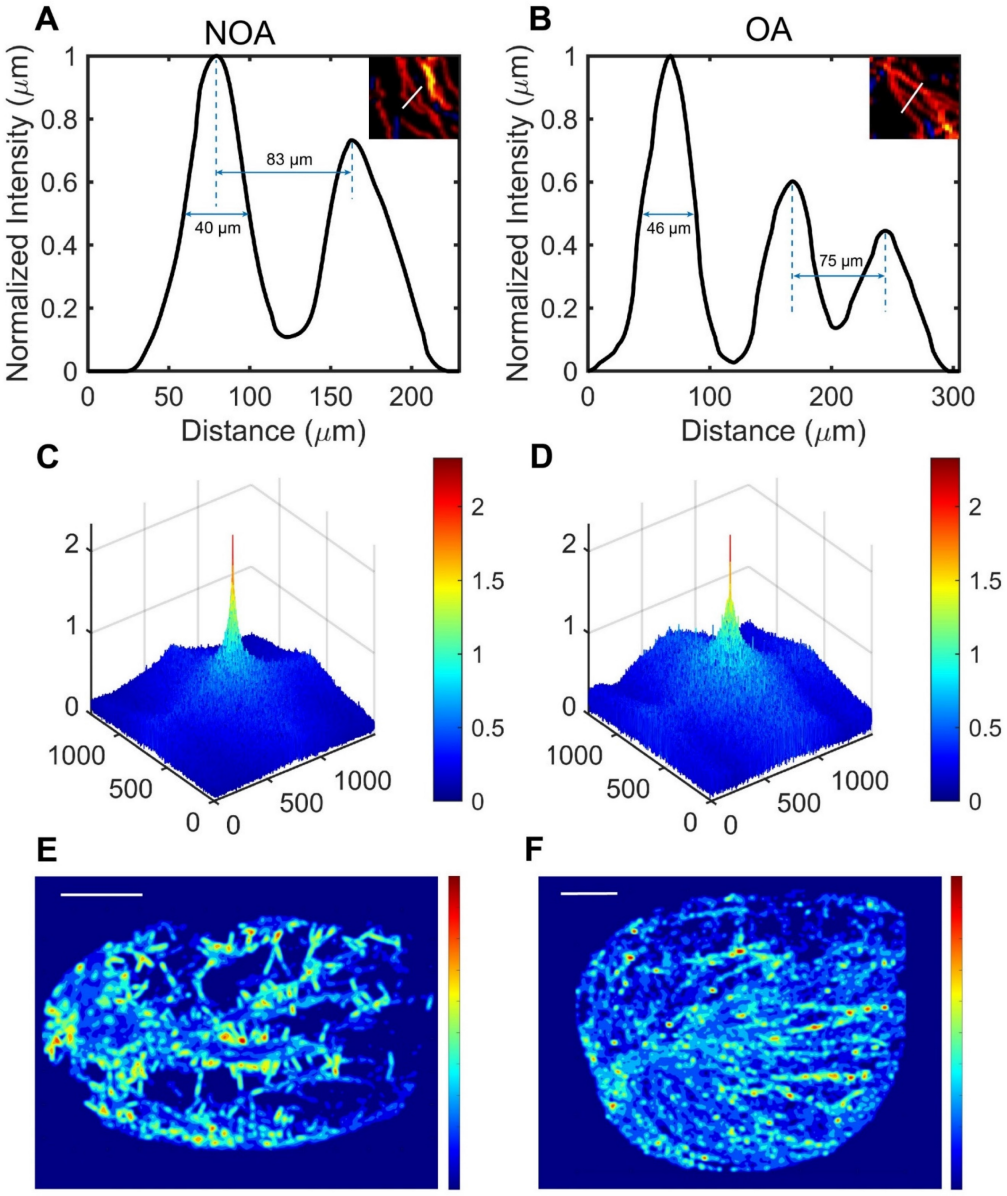
Comparison of testicular microcirculation manifestations in non-obstructive azoospermia (NOA) and obstructive azoospermia (OA) patients using ultrasound localization microscopy (ULM). (A) & (B) Normalized vessel intensity profiles along a selected distance for NOA and OA, respectively, with the selected distance marked by white lines in the magnified regions shown in Figure 3. (C) & (D) Frequency spectra generated using Fourier transform in NOA and OA. (E) & (F) Vascular pressure distribution maps derived from super-resolution velocity maps in NOA and OA testes. Scale bars = 5 mm.

Next, we magnified the same regions of the SR images in figure 3 for NOA and OA to demonstrate the ability of ULM to distinguish blood vessels. By comparing magnified images of the same regions, it was observed that OA patients had continuous vascular trajectories, more vascular branches, higher vascular density, and faster blood flow speeds. In Figure 4A, the normalized intensity cross-sectional profile of NOA showed two prominent peaks with a spacing of 83 µm. This inter-peak distance indicated the resolution, reflecting the ability to distinguish between two closely situated blood vessels. Similarly, in Figure 4B, adjacent microvessels that were 75 µm apart were clearly resolved. The width of each individual peak corresponded to the diameter of the measured blood vessel, which was 40 µm and 46 µm in NOA and OA, respectively.

We utilized Fourier transform to obtain the three-dimensional frequency spectra of NOA and OA (Figure 4C-D), providing critical insights into their vascular characteristics. The spectrum for NOA revealed a limited number of significant peaks, suggesting a less complex vascular structure with fewer microvessels. In contrast, the spectrum for OA was characterized by a higher density of signals and greater spectral complexity, and the high-frequency signal component of the OA frequency spectrum was higher. This indicated a more intricate microvascular network within OA tissues, with more microvessels, more image details, and a complex structure.

Pressure distribution maps were derived from SR velocity images, offering insights into the vascular pressure dynamics of NOA and OA. In Figure 4E, the pressure distribution showed a heterogeneous pattern with multiple regions of varying pressure. There were distinct high-pressure zones, indicated by red and yellow colors, scattered throughout the testis. These high-pressure areas were interconnected by regions of intermediate pressure (green and yellow), surrounded by low-pressure zones (blue). This suggested that the pressure distribution map for NOA was characterized by relatively uneven pressure levels with notable areas of pressure deficiency. This non-uniformity indicated disrupted or inconsistent blood flow. In contrast, the pressure distribution map for OA, displayed in Figure 4F, exhibits a heterogeneous pattern but differs from NOA in its organization. High-pressure zones (red and yellow) are more uniformly distributed along what appears to be directional pathways or linear structures. The intermediate pressure areas (green and yellow) are more continuous and less scattered compared to NOA. The overall pattern suggests a more organized structural characteristic in the testis compared to the one shown in NOA. To elucidate the potential relationship between the pressure distribution map of NOA and OA and their corresponding pathological conditions, we conducted a comparative analysis of the testicular histopathological slices from NOA and OA patients. Histopathological slices revealed that the testicular tissue of NOA patients exhibited hyalinization and atrophy of seminiferous tubules, arrested spermatogenic development, and absence of mature spermatozoa within the tubular lumina, interstitial fibrosis, and atrophy of surrounding blood vessels, indicative of degenerative pathological changes and decreased vascular pressure (Figure S2A-C). In contrast, in OA patients, histopathological findings showed the presence of spermatozoa, thickening of the peritubular wall with increased collagen deposition in the tubular basal lamina, accompanied by dilation of vascular lumina and congested peritubular capillaries (Figure S2D-F), associated with compensatory increases in blood flow and a more uniform distribution of vascular pressure within the testicular circulation.

### Quantitative Assessment of Hemodynamics in Azoospermic Testicular Microcirculation

**Figure 5:**
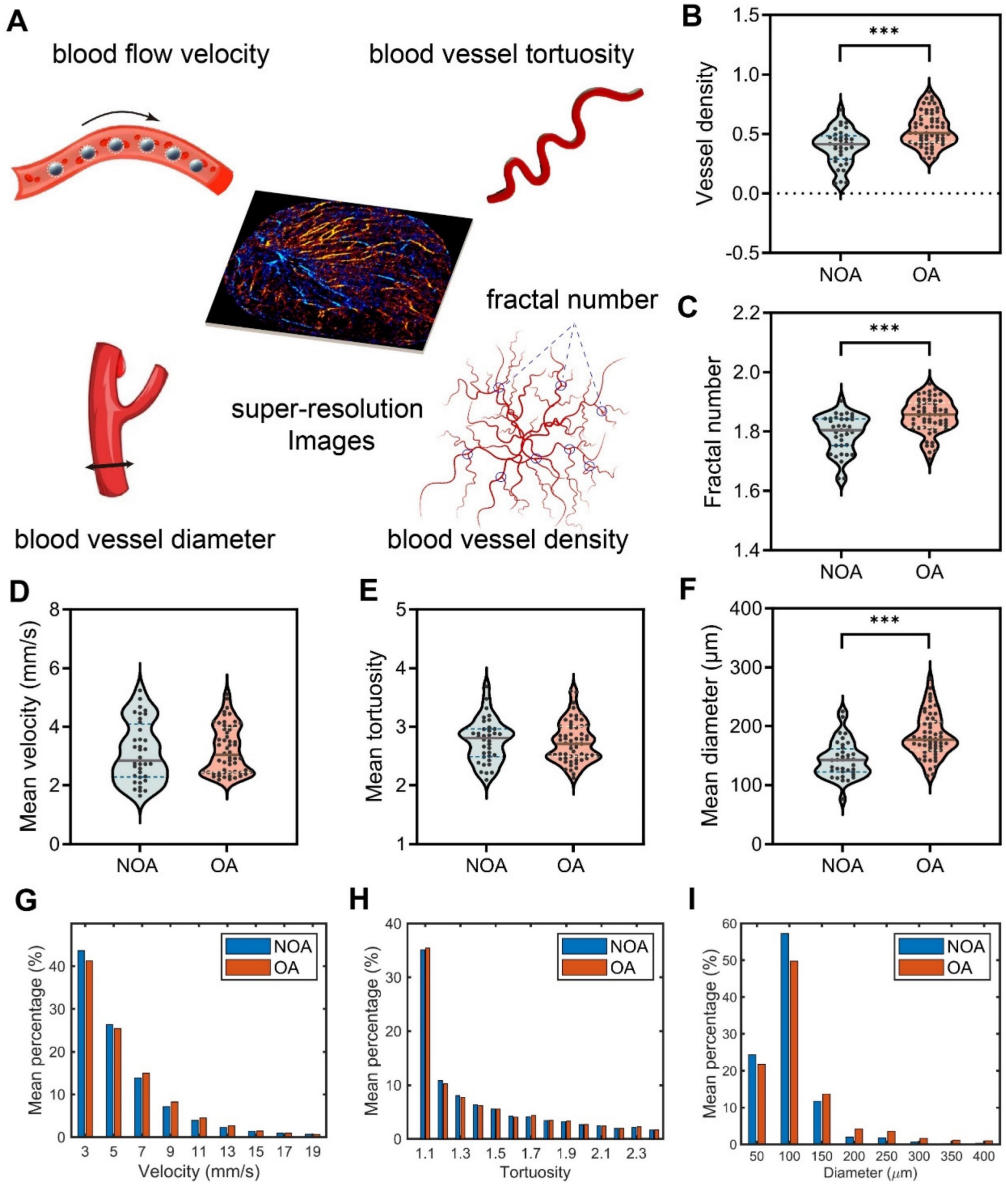
Quantitative assessment of hemodynamics in testicular microcirculation of all patients in non-obstructive azoospermia (NOA) and obstructive azoospermia (OA) groups. (A) Five vascular-related quantitative parameters were extracted from the SR images of the testes. (B-F) Violin plots depicting the distribution of vessel density, fractal number, mean velocity, mean tortuosity, and mean diameter of testicular microvasculature. The dashed lines represent quartiles, the solid lines represent the median, and the dots represent the individual values of each sample. Asterisks indicate statistically significant difference in means (results calculated as shown in Table 1). ^***^ indicates p < 0.001. (G-I) Histograms of the mean distribution of velocity, tortuosity, and diameter in the testes of all NOA and OA patients.

Next, in order to quantitatively compare the testicular microcirculation of NOA and OA, we extracted five vascular-related parameters from the SR images of the testes for quantitative analysis, including blood flow velocity, blood vessel tortuosity, blood vessel diameter, fractal number, and blood vessel density, as shown in Figure 5A. Mean velocity refers to the average rate of blood flow through the testicular vasculature. Tortuosity quantifies the degree of winding or curvature within a blood vessel, and mean tortuosity is the average measure of tortuosity across all vessels in a defined testicular vasculature. Mean diameter denotes the average internal width of the blood vessels within a defined vascular network of the testis. Fractal number serves as an index of the complexity and branching patterns of testicular blood vessels. It quantitatively measures how the vascular network fills space. A higher fractal number suggests a more intricate and densely branched vascular structure. Vessel density is defined as the number of blood vessels per unit area in the testicular microcirculation and serves as an indicator of tissue perfusion within the testis. The methodology for calculating these indicators is described in the “Methods” section. We calculated the mean values of all patients for analysis.

Table 1 presents a comparative analysis of the mean values of the vascular parameters of all patients within the NOA and OA groups. To further illustrate the distribution and variability of these parameters, violin plots are shown in Figure 5B-F. When comparing the five hemodynamic parameters between the NOA and OA groups, it was observed that the NOA group exhibited a slightly higher and more variable mean velocity compared to the OA group, but without statistical significance. Mean Tortuosity showed no significant differences. However, there were notable differences between the NOA and OA groups in terms of mean diameter (p < 0.001), vessel density (p < 0.001) and fractal number (p < 0.001), with the OA group consistently showing higher mean values and greater variability across all the three parameters. The histograms presented in Figure 5G-I provide a detailed comparative analysis of the mean distribution of blood flow velocity, vessel tortuosity, and vessel diameter within the testicular microcirculation of all patients in NOA and OA groups. The histograms corroborate our previous findings, showing that the NOA group exhibited a higher frequency of lower blood flow velocity, although this difference did not reach statistical significance. Both groups showed similar tortuosity distributions, aligning with the lack of significant differences in mean tortuosity. Additionally, the NOA group had a higher frequency of smaller vessel diameters, consistent with our earlier findings of significantly higher mean diameters in the OA group.

**Figure 6:**
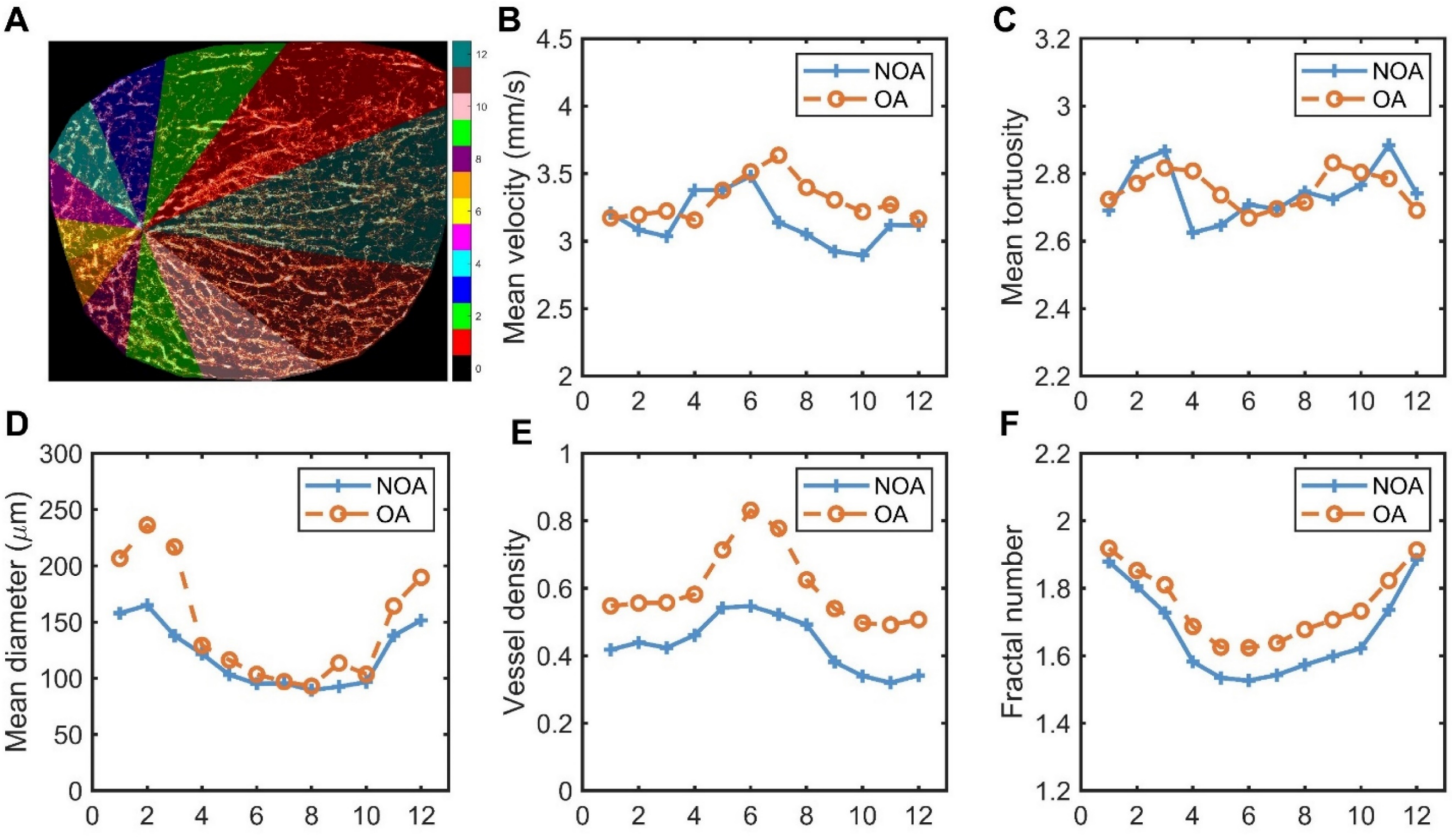
Schematic diagram of testicular regional division and quantitative comparison of microvascular parameters in each region for all non-obstructive azoospermia (NOA) and obstructive azoospermia (OA) patients. (A) Segmented regions of a testis, with different colors representing various zones (1-12) used for analysis, and the scale indicating the corresponding regions. (B-F) Graphs showing mean velocity, mean tortuosity, mean diameter, vessel density, and fractal number in different regions for NOA and OA.

The previous calculations were performed on the testis as a whole. Next, we segmented the testis into different regions to explore the differences in blood flow parameters within various regions of the testis in NOA and OA groups. We requested experienced andrologists to identify the position of the testicular hilum. Starting from this position and following the direction of the testicular long axis, the testicular image was divided into 12 regions in a counterclockwise direction, with each region encompassing 30° intervals. The schematic diagram of the partitioning method is shown in Figure 6A, highlighting different vascular regions using distinct colors. The 12 distinct regions corresponded to the 12 directions around the testicular vascular convergence hilum in the maximum transverse section. Figure 6B-F present the comparative analysis of various vascular parameters across these regions in all NOA and OA patients. Figure 6B-C indicate that both groups exhibited fluctuations in mean velocity and mean tortuosity values across all regions, with no significant overall differences between the NOA and OA groups. This suggests that the blood flow speed and degree of vessel curvature were relatively similar across different regions for both groups, despite local variations. The analysis of mean diameter, vessel density, and fractal number, depicted in Figure 6D-F, consistently showed that the OA group exhibited higher values compared to the NOA group across most regions. Figure 6D demonstrates that the OA group had larger mean vessel diameter, with the most significant differences observed in regions 1, 2, 11, and 12. These regions also correspond to the areas with the largest segmented areas of the testis, indicating wider blood vessels in these regions for the OA group. Figure 6E-F illustrate that the OA group had higher vessel density and fractal number, particularly in regions 5 to 8, which are the areas closest to the vascular convergence hilum. The higher vessel density in these regions suggests a denser network of blood vessels, while the increased fractal number indicates more complex and intricate vascular branching. The proximity to the hilum might contribute to the higher perfusion and complexity in these regions.

### Revelation of the Underlying Connection between Testicular Microcirculation and Spermatogenetic Functions in Azoospermia Patients

**Figure 7:**
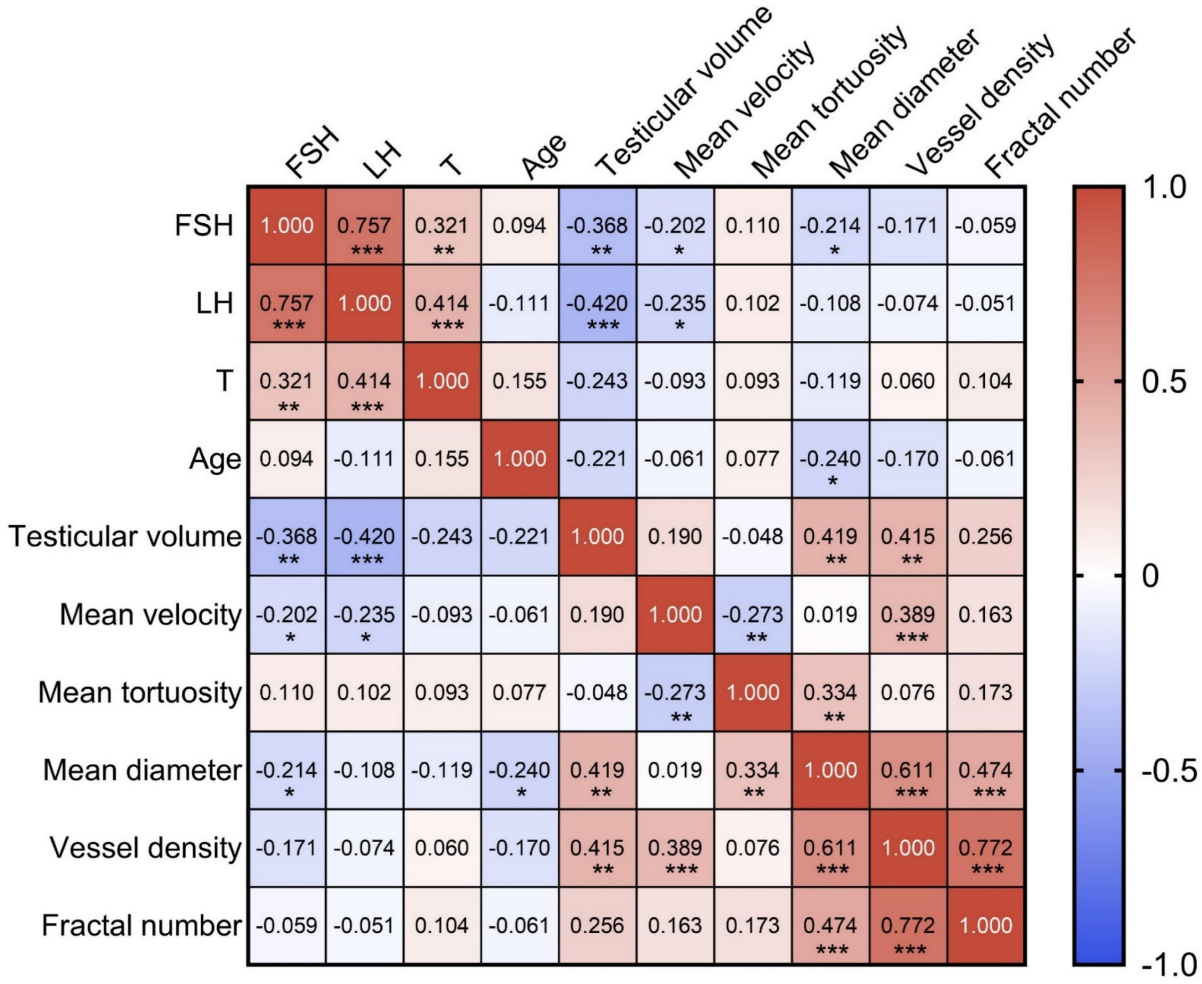
Heatmap of Pearson correlation coefficient matrix representing the relationships among various parameters of spermatogenic functions and testicular microcirculation in azoospermia patients. Each cell displays the Pearson correlation coefficient between pairs of variables, where 1.0 indicates a perfect positive correlation, 0 indicates no correlation, and -1.0 indicates a perfect negative correlation. The colors range from red (positive correlation) to blue (negative correlation). FSH: follicle-stimulating hormone; LH: luteinizing hormone; T: testosterone. ^***^ indicates p < 0.001, ^**^ indicates p < 0.01, ^*^ indicates p < 0.05.

Given the quantitative vascular parameters provided by ULM and its capability for detailed analysis, we have, for the first time, revealed the inner connection between testicular microcirculation and male spermatogenic functions. Clinical evaluation indicators of testicular spermatogenic functions include FSH, LH, T, age, and testicular volume. A Pearson correlation analysis was conducted between these five indicators and the aforementioned five ULM-derived vascular parameters (Figure 7).

#### Correlations Between the Clinical Indicators of Spermatogenic Functions

FSH and LH show a strong positive correlation (r = 0.757, p < 0.001), suggesting a synchronous regulatory effect on testicular functions. Both FSH and LH exhibit negative correlations with testicular volume, with correlation coefficients of -0.368 (p < 0.01) and -0.420 (p < 0.001) respectively, indicating that higher hormone levels are associated with smaller testicular size. T shows a moderate positive correlation with LH (r = 0.414, p < 0.001) and a weaker one with FSH (r = 0.321, p < 0.01). Age is poorly correlated with all other measured clinical indicators, suggesting that age might not significantly influence these specific testicular and hormonal metrics within the sampled population.

#### Correlations Between Vascular Parameters of Testicular Microcirculation

The relationship between mean velocity and vessel density reveals a statistically significant moderate positive correlation (r = 0.389, p < 0.001). This suggests that regions with higher blood flow speeds are adapted to have a greater number of blood vessels. Moreover, there is a weak negative correlation between mean velocity and mean tortuosity (r = -0.273, p < 0.01), implying that faster blood flow might be facilitated by less tortuous vessels. The data also indicate a weak positive correlation between mean tortuosity and mean diameter (r = 0.334, p < 0.01), suggesting that vessels with greater tortuosity, or more twists and turns, tend to have slightly larger diameters.

More pronounced relationships are observed in correlations involving mean diameter, vessel density, and fractal number. Mean diameter and vessel density show a moderate positive correlation (r = 0.611, p < 0.001), indicating that areas with wider vessels also tend to have a higher concentration of testicular blood vessels. Similarly, the correlation between mean diameter and fractal number is also positive (r = 0.474, p < 0.001), suggesting that wider vessels are associated with more complex vascular branching, as indicated by a higher fractal number.

Lastly, a particularly notable positive correlation is observed between vessel density and fractal number (r = 0.772, p < 0.001). This high correlation highlights that regions with a higher density of vessels typically exhibit more complex microvascular architectures characterized by intricate branching patterns.

#### Correlations Between Spermatogenic Functions and Testicular Microcirculation

Testicular volume and mean diameter show a moderate positive correlation (r = 0.419, p < 0.01), suggesting that an increase in testicular volume corresponded to an enlargement in mean diameter. Similarly, a moderate positive correlation is also observed between testicular volume and vessel density (r = 0.415, p < 0.01), implying that the enlargement of testicular volume is associated with an enhanced microcirculatory system.

The negative correlation between FSH and mean diameter (r = -0.214, p < 0.05) reflects that increases in FSH levels are correlated with a decrease in testicular microvascular structural size in azoospermia patients. The negative correlations between FSH and mean velocity (r = -0.202, p < 0.05) and between LH and mean velocity (r = -0.235, p < 0.05) indicate that elevated FSH and LH levels might inversely affect the velocity parameters of the testicular microcirculation. The negative correlation between age and mean diameter (r = -0.240, p < 0.05) indicates that the diameter of testicular microvasculature decreases with age.

### Establishment of Diagnostic Models for Azoospermia Patients

**Figure 8:**
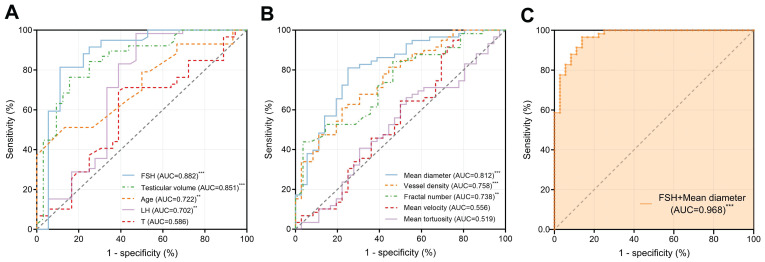
Receiver Operating Characteristic (ROC) curves demonstrating the diagnostic performance of different parameters in distinguishing between NOA and OA. (A) Clinical parameters: FSH, testicular volume, age, LH, and T. (B) Ultrasound localization microscopy (ULM)-derived vascular parameters: mean diameter, vessel density, fractal number, mean tortuosity, and mean velocity. (C) A multiple logistic regression model combining FSH and mean diameter showed the highest accuracy, with an AUC of 0.968. FSH: follicle-stimulating hormone; LH: luteinizing hormone; T: testosterone; AUC: area under the curve. ^***^ indicates p < 0.001, ^**^ indicates p < 0.01.

We constructed ROC curves to evaluate the diagnostic value of different spermatogenic indicators, including FSH, LH, T, age, and testicular volume, in distinguishing between patients with NOA and OA as shown in Figure 8A. The AUCs were 0.882 [95% confidence interval (CI): 0.799-0.965, p < 0.001], 0.702 (95% CI: 0.578-0.827, p < 0.01), 0.586 (95% CI: 0.465-0.707, p = 0.162), 0.722 (95% CI: 0.607-0.837, p < 0.01), and 0.851 (95% CI: 0.759-0.943, p < 0.001), respectively.

The discrimination abilities of ULM-derived vascular parameters, including mean velocity, mean tortuosity, mean diameter, fractal number, and vessel density, were also evaluated (Figure 8B). The AUCs were 0.556 (95% CI: 0.430-0.682, p = 0.361), 0.519 (95% CI: 0.397-0.641, p = 0.759), 0.812 (95% CI: 0.721-0.902, p < 0.001), 0.738 (95% CI: 0.629-0.846, p < 0.01), and 0.758 (95% CI: 0.661-0.855, p < 0.001), respectively.

We calculated the optimal cutoff values for the statistically significant parameters within the sampled population. The optimal cutoff value for FSH was 7.12 IU/L, with a sensitivity of 81.4% and a specificity of 88.9%. For testicular volume, the optimal cutoff value was 10.57 mL, with a sensitivity of 76.3% and a specificity of 84.4%. For age, the optimal cutoff value was 30 years, with a sensitivity of 51.2% and a specificity of 86.7%. For LH, the optimal cutoff value was 6.55 IU/L, with a sensitivity of 98.3% and a specificity of 52.8%. For mean diameter, the optimal cutoff value was 155.9 μm, with a sensitivity of 81.0% and a specificity of 75.0%. For vessel density, the optimal cutoff value was 0.489, with a sensitivity of 61.0% and a specificity of 77.8%. For fractal number, the optimal cutoff value was 1.863, with a sensitivity of 43.9% and a specificity of 96.4%.

We then combined two parameters that indicated spermatogenic functions and testicular microvasculature with the optimal diagnostic performances, respectively (Figure 8C). Combining FSH and mean diameter improved the diagnostic value, with an AUC of 0.968 (95% CI: 0.938-0.999, p < 0.001). The results of this multiple logistic regression diagnostic model are listed in Table 2. The model successfully classified 31 out of 36 NOA cases and 56 out of 58 OA cases, reflecting a specificity of 86.1% and a sensitivity of 96.6%, respectively. Overall, the accuracy of this model reached 92.6%. The negative predictive value of 93.9% indicates that the likelihood of a case predicted as NOA actually being NOA was very high, and similarly, the positive predictive value of 91.8% shows that cases predicted as OA were likely to be correctly identified. Additionally, the Pseudo R² value of 0.736 suggests that the model has a strong ability to explain the variability in the observed outcomes, indicating robust diagnostic power.

## Discussion

This study provides significant insights into the differentiation of NOA and OA using ULM technology. We performed ULM of the testes in NOA and OA patients, achieving a resolution of 75 µm, and obtained SR microvascular distribution maps and hemodynamic distribution maps. Our findings highlight several novel contributions to the field, notably the revelation of the inner connection between clinical indicators of spermatogenic functions and testicular microcirculation, the establishment of a high-performance diagnostic model for distinguishing NOA from OA, and the introduction of vascular pressure distribution maps.

For the first time, this study introduces pressure distribution maps derived from SR velocity distribution data, realizing the visualization and analysis of the hemodynamic environment within the testicular microvasculature from a new dimension. These innovative maps provide a new approach to understanding the intricate dynamics of blood flow and pressure within the testes of NOA and OA patients, offering a detailed representation of how blood velocity translates into vascular pressure. Specifically, regions exhibiting higher blood flow velocity corresponded to areas of increased vascular pressure. In NOA patients, the pressure maps demonstrated regions with uneven pressure distribution and areas of low or absent pressure, correlating with the observed sparse blood perfusion and lower vascular density. This suggests that the hemodynamic environment in NOA is characterized by regions of low pressure, potentially contributing to the compromised testicular functions seen in these patients. The regions of missing pressure correlated with the simpler and less dense vascular structure seen in NOA, suggesting a less robust blood supply, further exacerbating the already impaired spermatogenic functions 1, 31, 32. Conversely, in OA patients, the pressure distribution maps indicated more uniform vascular pressures throughout the testicular microvasculature. The uniform vascular pressures in OA suggest a more vigorous blood flow and better-maintained microcirculatory function, which aligns with the generally better-preserved testicular architecture in these patients 33, 34.

The pressure distribution maps derived from SR velocity distribution data provide valuable insights into the different underlying pathological conditions of NOA and OA. Our histopathological analysis indicated that NOA patients demonstrated degenerative alterations, including seminiferous tubular hyalinization, interstitial fibrosis, and vascular atrophy. The degenerative changes and vascular atrophy observed in NOA are consistent with the information conveyed by the pressure distribution maps, which reveal missing regions of pressure in NOA, leading to hypoperfused regions and impaired spermatogenesis. Conversely, OA patients exhibited peritubular thickening, increased collagen deposition in the tubular basal lamina, and vascular dilation and congestion. This phenomenon can be explained by the pathogenesis of OA. OA is characterized by a physical blockage in the male reproductive tract, resulting in increased resistance within the testicular and epididymal ducts, which leads to a buildup of pressure proximal to the obstruction 35, 36. This increased pressure in the epididymal and seminiferous tubules results in thickening of the tubular basal lamina, affects the microcirculatory environment within the testes, and consequently leads to a compensatory increase in blood supply and thickening of the microvascular network, consistent with relatively uniform vascular pressure to ensure nutrient supply to the seminiferous tubules. These pathological findings are also consistent with the histological features of NOA and OA previously reported in the literature 37.

The SR vascular and pressure distribution maps for NOA and OA are consistent with the phenomena observed in CDFI and CEUS imaging. Specifically, CDFI indicated that OA patients exhibited slightly more abundant testicular blood flow signals compared to NOA patients. CEUS imaging at the peak of MB perfusion revealed that testicular vascular perfusion was less dense in NOA patients than in OA patients. These observations align with the pathological mechanisms of the two conditions. The testicular histopathology of NOA is characterized by a series of degenerative changes, manifested in the testicular blood flow as localized hypoperfused regions with missing areas of vascular pressure. In contrast, the histopathology of OA patients, marked by dilation of vascular lumina and congested peritubular capillaries, corresponds to compensatory increased blood flow and relatively uniform vascular pressure in the testicular blood flow.

After obtaining the SR images, we proceeded with the quantitative analysis of the ULM-derived vascular parameters for all NOA and OA patients. Initially, through the analysis of the entire testis, we found that OA had higher mean diameter, vessel density, and fractal number, indicating more robust and complex vascular structures. To gain deeper insights, we conducted a regional analysis in 12 directions to explore the differences in blood flow parameters within various regions of the testes in NOA and OA groups. The analysis of these regions revealed several key differences. We found that OA consistently exhibited higher values in mean diameter, vessel density, and fractal number across most regions. Notably, OA had larger vessel diameters in regions corresponding to the largest segmented areas of the testis. This suggests that wider blood vessels in OA might be a compensatory mechanism to ensure adequate blood flow despite physical blockages in the reproductive tract 37. Additionally, higher vessel density and increased fractal number in OA, particularly near the vascular convergence hilum, suggested a denser and more complex vascular network. This could facilitate better microcirculatory function and more efficient blood distribution, contributing to the generally better-preserved testicular architecture observed in OA patients.

Another groundbreaking aspect of our study is the revelation of the underlying connection between spermatogenic functions and testicular microcirculation in azoospermia patients. In the human testis, capillaries serially connect Leydig cells and seminiferous tubules, playing a critical role in spermatogenesis by facilitating hormone transport and nutrient supply. The presence of androgen and estrogen receptors on these capillaries underscores their involvement in the endocrine and paracrine regulation of sperm production. Specifically, androgen receptors located on arterial and intramural capillaries suggest that androgens may modulate the blood supply essential for the function of Leydig cells and seminiferous tubules, highlighting the testicular microvasculature's key role in supporting reproductive health 11, 38-40. In our previous work, we confirmed the feasibility of ULM for visualizing testicular microcirculation and utilized the ULM-derived vascular parameters to differentiate between obstructive and non-obstructive etiologies 41. However, we did not investigate the direct connection between testicular microcirculation and spermatogenic functions. In this study, we have addressed this aspect.

The quantitative vascular parameters derived from ULM, including mean velocity, mean tortuosity, mean diameter, fractal number, and vessel density, were found to correlate significantly with clinical indicators such as FSH, LH, testosterone, age, and testicular volume 42-47. The observed significant correlations between spermatogenic function indicators and testicular microcirculation parameters can be explained through underlying physiological mechanisms. The moderate positive correlation between testicular volume and both mean diameter (r = 0.419, p < 0.01) and vessel density (r = 0.415, p < 0.01) suggests that larger testes require an enhanced microcirculatory system to support their functions. This relationship ensures that larger testes receive sufficient blood supply to maintain spermatogenesis. FSH is generally associated with promoting the division and maturation of spermatogenic cells, and in azoospermia patients, higher FSH levels were found to correlate negatively with mean diameter (r = -0.214, p < 0.05), indicating that elevated FSH may reflect compromised microvascular structures. LH, another hormone closely related to gonadal function, also showed a negative correlation with mean velocity (r = -0.235, p < 0.05), suggesting that elevated LH levels might be associated with impaired blood flow. These hormonal influences highlight the critical role of endocrine regulation in maintaining testicular microcirculation. We also observed a negative correlation between age and mean diameter (r = -0.240, p < 0.05), reflecting reduced blood vessel diameter with aging, which is related to the natural decline in spermatogenic functions as a result of physiological aging.

Additionally, one of the most significant contributions of this study is the establishment of a high-performance diagnostic model for differentiating NOA from OA by combining clinical spermatogenic function indicators with testicular microcirculation parameters. Individually, clinical parameters such as FSH and testicular volume demonstrated great diagnostic value, with AUCs of 0.882 and 0.851, respectively. Similarly, microcirculatory parameters like mean diameter and vessel density showed good discriminatory power, with AUCs of 0.812 and 0.758, respectively. However, a substantial improvement was observed in diagnostic accuracy when these parameters were combined. The model, which integrated FSH and mean diameter, demonstrated exceptional diagnostic accuracy with an AUC of 0.968 (95% CI: 0.938-0.999, p < 0.001), significantly enhancing the performance compared to the use of individual parameters alone. This combined approach achieved a specificity of 86.1%, a sensitivity of 96.6%, a negative predictive value of 93.9%, a positive predictive value of 91.8%, and an overall accuracy of 92.6%, demonstrating its robustness and reliability in distinguishing between NOA and OA. The integration of the clinical parameter and the microcirculatory parameter provides a more comprehensive assessment of testicular function. The clinical parameter reflects the overall hormonal environment and its regulatory effects on spermatogenesis, while the microcirculatory parameter offers detailed insights into the microvascular structures that support testicular function. The significant correlations found between FSH and mean diameter in azoospermia mentioned above further support their combined use. By combining these datasets, the model captures a broader spectrum of pathophysiological changes, enhancing diagnostic precision.

However, the study has some limitations. While our research provides initial insights into testicular microcirculation and spermatogenic functions based on cross-sectional data, it does not capture how these dynamics might evolve over time with medical interventions or disease progression. The study also focuses mainly on basic clinical parameters like FSH, LH, and T, omitting a broader range of potentially meaningful biomarkers such as anti-Müllerian hormone (AMH) 48, 49, which could provide a more comprehensive understanding of the biological underpinnings and potentially uncover other factors that may influence the accuracy of diagnosis. Additionally, while ULM offers detailed visualization, it is limited to two-dimensional planes of the testes, which does not fully reflect the complex three-dimensional nature of the testicular environment. Implementing three-dimensional ULM could offer a more complete and accurate representation of testicular structure and function. Looking ahead, ULM technology could also play a pivotal role in guiding micro-testicular sperm extraction (mTESE) procedures by providing detailed insights into testicular microcirculation, which can inform sperm retrieval decisions and prognostic assessments 50-52. These areas underscore the need for further research to refine the findings and enhance their clinical applicability, particularly in terms of diagnostic accuracy and therapeutic guidance. Future research should also focus on validating these findings in larger, diverse cohorts and exploring the broader applicability of these techniques in other testicular pathologies.

## Conclusion

In conclusion, this study underscores the transformative potential of ULM in the differentiation diagnosis of NOA and OA. By uncovering the inner connection between testicular microcirculation and clinical spermatogenic functions, establishing a high-performance diagnostic model and reporting a method to map the pressure distribution, we have paved the way for more precise and effective diagnostics in male infertility. The ability to accurately distinguish between NOA and OA has crucial implications for treatment planning and patient management, potentially leading to more personalized and effective therapeutic strategies. This study not only enhances our understanding of azoospermia but also ays the groundwork for future innovations in reproductive health diagnostics.

## Supplementary Material

Supplementary figures.

## Figures and Tables

**Table 1:** Comparison of Hemodynamic Parameters in Azoospermia

Parameters	NOA (n = 36)	OA (n = 58)	p value
Mean velocity (mm/s)	3.130 (1.024)	3.214 (0.813)	0.663
Mean tortuosity	2.759 (0.368)	2.742 (0.343)	0.815
Mean diameter (μm)	143.1 (31.3)	183.6 (37.1)	<0.001^***^
Vessel density	0.392 (0.148)	0.542 (0.143)	<0.001^***^
Fractal number	1.793(0.063)	1.852 (0.057)	<0.001^***^

The values in the table were means, with standard deviations in parentheses. NOA: non-obstructive azoospermia; OA: obstructive azoospermia. ^***^ indicates p < 0.001.

**Table 2:** Multiple Logistic Regression Diagnostic Model for Azoospermia Differentiation

Variable	β	95% CI for β	SE	OR	95% CI for OR	p value
(Intercept)	-8.773	(-15.990, -3.508)	3.122	1.549×10^-4^	(1.133×10^-7^, 0.030)	0.0050^**^
FSH	-0.833	(-1.376, -0.479)	0.224	0.435	(0.253, 0.619)	0.0002^***^
Mean diameter	0.089	(0.049, 0.150)	0.025	1.094	(1.050, 1.162)	0.0004^***^
		Predicted				
Observed	NOA	OA	Total	Correctly classified		
NOA	31	5	36	86.1%	Negative predictive value	93.9%
OA	2	56	58	96.6%	Positive predictive value	91.8%
Total	33	61	94	92.6%	Pseudo R^2^	0.736

The values in parentheses represent the lower and upper bounds of 95% confidence interval. 95% CI: 95% confidence interval; β: coefficient; SE: standard error; OR: odds ratio; FSH: follicle-stimulating hormone; NOA: non-obstructive azoospermia; OA: obstructive azoospermia. Negative predictive value indicates the proportion of cases predicted as NOA that were actually NOA, and positive predictive value indicates the proportion of cases predicted as OA that were actually OA. ^***^ indicates p < 0.001, ^**^ indicates p < 0.01. The classification cut off value was 0.5.

## Abbreviations

AMH: anti-Müllerian hormone
AUC: area under the curve
CEUS: contrast-enhanced ultrasound
CI: confidence interval
CDFI: color Doppler flow imaging
FSH: follicle-stimulating hormone
LH: luteinizing hormone
MIP: maximum intensity projection
MB: microbubble
NOA: non-obstructive azoospermia
OA: obstructive azoospermia
ROI: region of interest
SD: standard deviation
SR: super-resolution
SVD: singular value decomposition
T: testosterone
ULM: ultrasound localization microscopy
mTESE: micro-testicular sperm extraction
