# Supplementary material for: Non-invasive ultrasound localization microscopy (ULM) in azoospermia: connecting testicular microcirculation to spermatogenic functions
Source: Theranostics. 2024 Aug 19;14(13):4967–82. doi: 10.7150/thno.99668 (PMC11388075; doi:10.7150/thno.99668)
Supplement: Supplementary file 1 — Supplementary figures. [file thnov14p4967s1.pdf]

Supplementary Material

Non-invasive ultrasound localization microscopy (ULM) in azoospermia: connecting testicular microcirculation to spermatogenic functions

Maoyao Li[1#], Xingxuan Zhang[1#], Jipeng Yan[2], Huiquan Shu[3,4], Zitong Li[1], Chujun Ye[1], Lei Chen[1], Chao Feng[3,4*], Yuanyi Zheng[1*]*

Maoyao Li and Xingxuan Zhang contributed equally to this work.

1 Department of Ultrasound in Medicine, Shanghai Sixth People's Hospital Affiliated to Shanghai Jiao Tong University School of Medicine, Yishan Road 600, Shanghai, 200233, China.

2 Department of Bioengineering, Imperial College London, Exhibition Road, London, SW7 2AZ, U.K.

3 Department of Reproductive Medicine, The International Peace Maternity and Child Health Hospital, School of Medicine, Shanghai Jiao Tong University, Shanghai, 200030, China

4 Shanghai Key Laboratory of Embryo Original Disease, Shanghai 200030, China

***Corresponding Authors**

Email: andrewch@263.net; fengchao9901790@aliyun.com; zhengyuanyi@sjtu.edu.cn

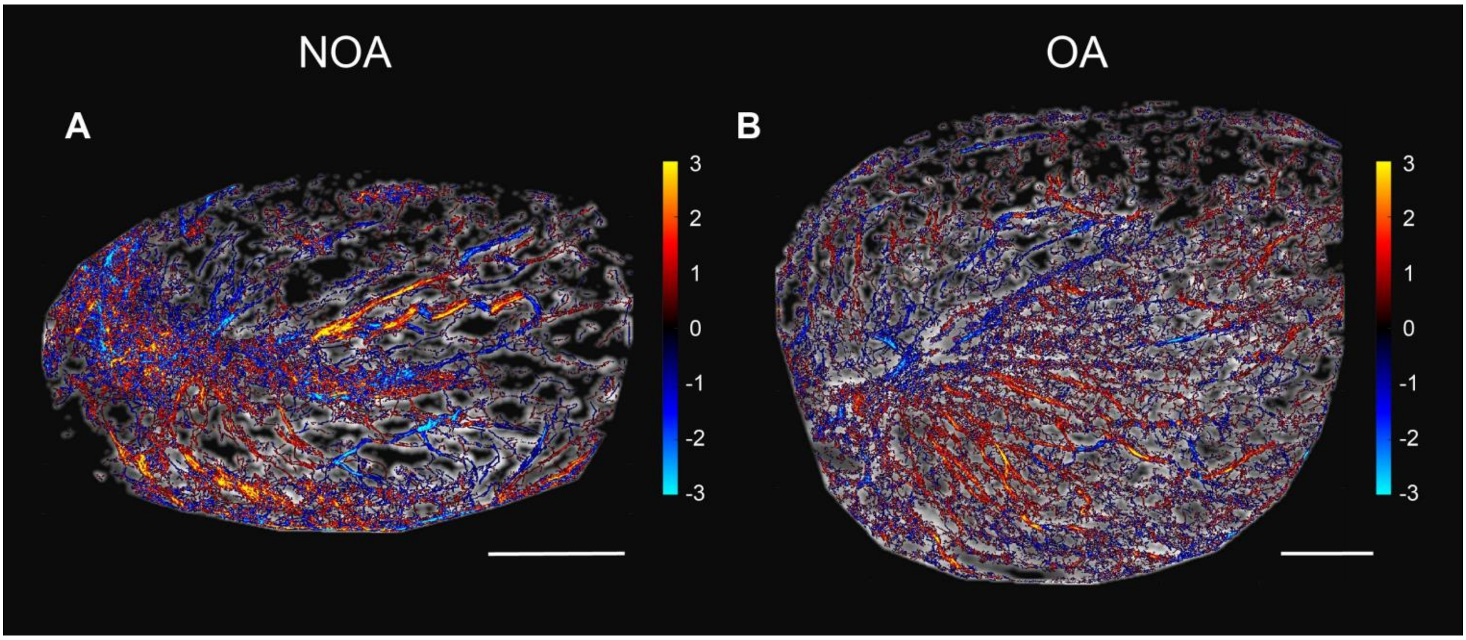

Figure S1. Superimposed ultrasound localization microscopy (ULM) super-resolution (SR) images of non-obstructive azoospermia (NOA) and obstructive azoospermia (OA) patients with their corresponding MIP images. (A) shows the image for NOA, while (B) shows the image for OA. Scale bars = 5 mm.

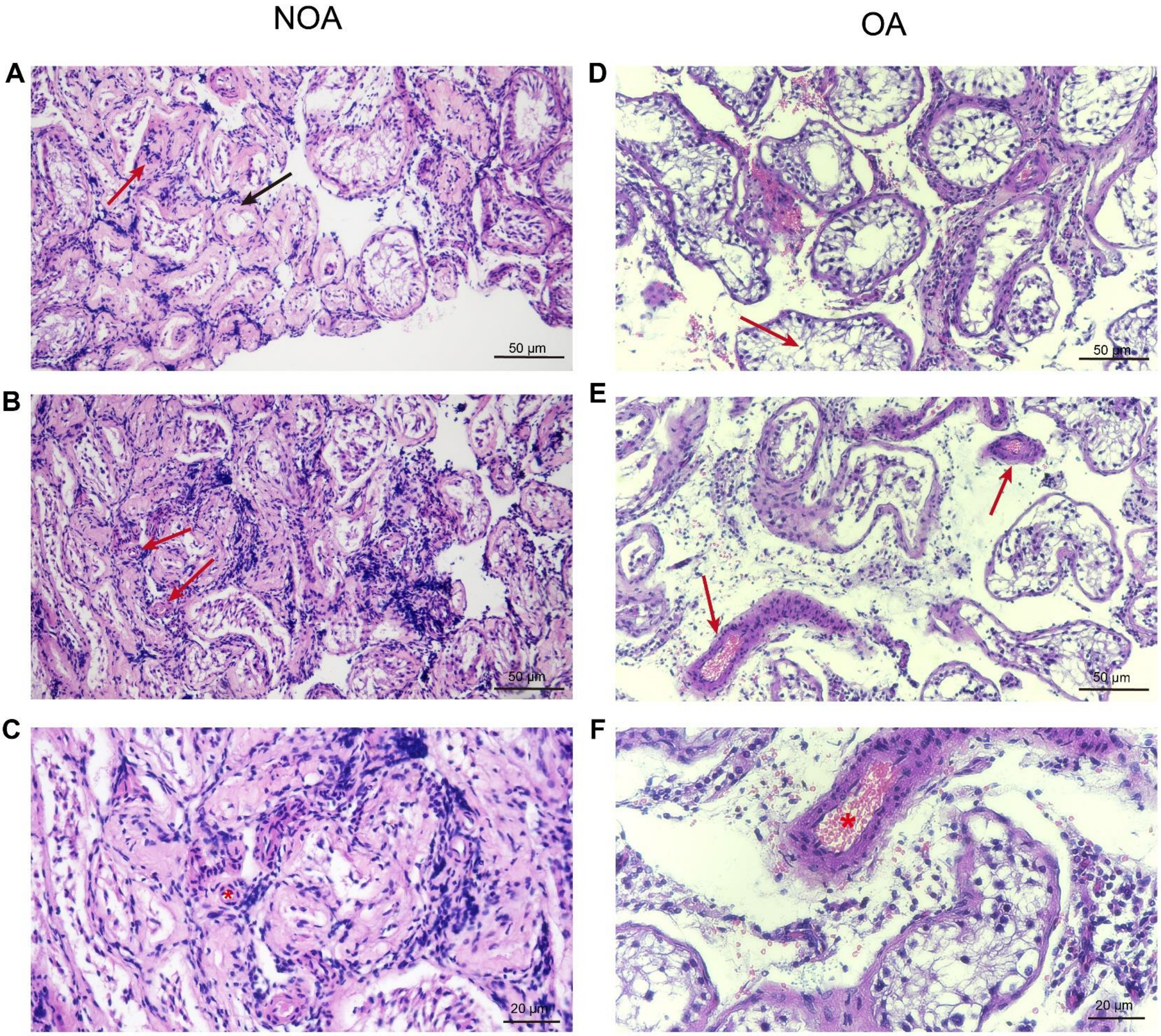

Figure S2. Histopathological analysis of testicular tissue in non-obstructive azoospermia (NOA) and obstructive azoospermia (OA) patients. Panels A-C represent NOA, while panels D-F represent OA. (A) The red arrow indicates interstitial fibrosis, and the black arrow shows hyalinization of the seminiferous tubules. (B) The two red arrows pointed to atrophic blood vessels in NOA, with one of them marked by an asterisk in (C) at a magnification of ×20. (D) The red arrow identifies a spermatozoon. (E) The two red arrows highlight congested and dilated blood vessels in OA, with one of them marked by an asterisk in (F) at a magnification of ×20. The magnification for the other images is ×10.
